# A literature-informed Delphi framework for pediatric medication science books

**DOI:** 10.3389/fped.2026.1873699

**Published:** 2026-08-26

**Authors:** Jing Xie, Wenxu Sun, Siying Wang, Xia Yang, Lanting Yao

**Affiliations:** 1 Department of Pharmacy, Institute of Clinical Pharmacy, West China Hospital of Sichuan University, Chengdu, Sichuan, China; 2 West China School of Clinical Medicine, Sichuan University, Chengdu, China; 3 West China School of Pharmacy, Sichuan University, Chengdu, China

**Keywords:** book content framework, Delphi method, indicator system, literature research, pediatric science popularization

## Abstract

**Objective:**

To construct a quantifiable indicator system for evaluating pediatric pharmacology knowledge and to provide a scientific basis for writing popular science books on pediatric medication.

**Methods:**

From June to July 2025, a systematic literature search was performed to conduct a general systematic review of pediatric medication science popularization content. Literature information was integrated via thematic synthesis to formulate a preliminary questionnaire. Experts from tertiary hospitals and colleges of pharmacy across eastern, central and western China were selected, and a single-round modified Delphi method was carried out by distributing and collecting questionnaires through an online survey platform (Wenjuanxing). A dual-threshold criterion was applied for indicator screening: items with a mean importance score ≥ 4.0 and a coefficient of variation (CV) ≤ 25% were directly included. For items failing to meet the above criteria, the research team conducted joint deliberation combined with experts' open-ended qualitative feedback, to determine whether the items would be excluded, or revised and merged into relevant retained items.

**Results:**

A total of 3,520 studies were initially retrieved, yielding 68 included studies that generated 11 first-level and 54 s-level indicators. Of 28 distributed questionnaires, 26 were valid, yielding a valid response rate of 92.86%. The mean importance scores for the 11 first-level indicators ranged from 3.49 to 4.67, with all CVs ≤25% (11.89%–22.53%). Indicators with an intraclass correlation coefficient (ICC) at a moderate or higher level accounted for 90.9%. Ultimately, 6 first-level indicators met the dual-threshold criteria and were retained. “*in vivo* Drug Process” was retained following team discussion, while “Prescription Reading” and “Drug Economy and Regulation” were excluded due to abstractness and low scores. “Drug Efficacy and Toxicity” and “Drug Use” were merged into “Medication Safety” and “Home Medication Guide,” respectively.

**Conclusion:**

The pediatric medication knowledge framework indicator system established based on the systematic literature review and single-round modified Delphi consultation has a high degree of expert consensus and a clear screening process, with sound authority, scientificity and operability, providing a theoretical basis and practical reference for the content design of general pediatric medication science popularization books.

## 1 Introduction

With the advancement of the “Healthy China” strategy and the growing societal awareness of medication safety, providing systematic and scientific pharmaceutical education to children and adolescents has become an urgent social need (1). Studies have shown that there is still considerable room for improvement in the cognitive level of children and their guardians regarding safe medication knowledge (2), especially misunderstandings in dealing with missed doses, dosage, timing of drug withdrawal, self-purchase of drugs, and handling of adverse reactions (3), which directly lead to tens of thousands of adverse drug events in children every year (4, 5). However, the current work of pediatric pharmaceutical science popularization in China is faced with three dilemmas: a serious shortage of high-quality popular science resources, a lack of professional creative teams, and the absence of an evaluation standard system.

Existing popular science works generally have problems such as fragmented content, overly professional expression, and lack of age-appropriate design, which make it difficult to effectively cater to children's cognitive development level and reading interests, thus restricting the actual effectiveness of science popularization education (6). Internationally, developed countries have long included the cultivation of children's health literacy into national strategies. For example, the American Academy of Pediatrics (AAP) has issued Guidelines for Safe Medication Use in Children (7), the European Union has formulated Standards for Pediatric Pharmaceutical Science Popularization (8), and Japan has established a systematic “pharmacy education” curriculum system (9).

Effective science popularization education should not only stay in the one-way instillation of knowledge; its deeper goal is to cultivate core literacy that can benefit children for a lifetime, including multi-dimensional abilities such as scientific cognition, information screening, safe practice, and innovative spirit (10, 11). Guided by this concept, this study screened and constructed a key indicator system applicable to the content framework of general pediatric medication science popularization books through systematic literature research and the single-round modified Delphi method. With children as the direct target audience, this framework integrates “family collaboration” scenarios (e.g., household medicine management) into its indicator design, aiming to in turn drive improvements in guardians' medication practices through the enhancement of children's cognition.

The indicators of this system are not an isolated list of knowledge points, but closely aligned with specific educational goals and literacy cultivation dimensions. It aims to provide a clear logical path and scientific basis for the design, organization and optimization of popular science book content, thereby laying a foundation for the standardized and systematic development of pediatric medication science popularization, and supporting the in-depth implementation of the “Healthy China” strategy in the field of children's health literacy.

## 2 Materials and methods

### 2.1 General systematic review of pediatric pharmaceutical science popularization content

#### 2.1.1 Inclusion criteria

In this study, the inclusion criteria were established in accordance with the methodological framework of Systematic Review and the Population-Concept-Context (PCC) principle, as specified below: (1) Population: Children aged 0–18 years, their parents or guardians, and educators (including school teachers, health education personnel, etc.). (2) Concept: (a) Standards, guidelines, specifications and popular science materials for pediatric medication science popularization (including books, picture books, audio-video materials, course lesson plans, etc.); (b) Current cognitive status of pediatric medication safety knowledge, information access channels, and preferences for popular science content among parents, guardians and educators; (c) Current prevalence and influencing factors of drug misuse, drug abuse and adverse drug events. (3) Context: Settings where the studies were conducted or applied include but are not limited to schools (kindergartens, primary and secondary schools), communities, medical institutions (hospitals, community health service centers), home environments, and online health information platforms. (4) Literature types: Published journal articles, academic theses and dissertations, conference abstracts with complete data, standard documents, as well as technical reports, guidelines and position papers issued by government authorities or professional academic societies were eligible for inclusion.

#### 2.1.2 Exclusion criteria

(1) Repeatedly published literature; (2) Literature with incomplete original data or questionnaire structure; (3) Literature with unavailable full text; (4) Literature with ineligible publication types; (5) Literature with irrelevant content; (6) Duplicately included review literature; (7) Literature not published in Chinese or English.

#### 2.1.3 Search strategy

Research studies were systematically retrieved from CNKI, Wanfang Data Knowledge Service Platform, VIP Network, SinoMed, PubMed, Embase, and Cochrane Library, as well as standard studies from the National Standard Information Public Service Platform, National Standard Library, CNKI Standard Library, International Organization for Standardization, American National Standards Institute, and European Standards, with supplementary retrieval from Baidu and Bing. To focus on the latest research progress and policy developments in this field over the past five years, the retrieval period was set from January 2020 to May 2025. A systematic search was conducted using a combination of MeSH terms and free-text words in titles and abstracts. The search terms for **population** included: Child, Children, Adolescent, Pediatric Patient, Minor, Parent, Guardian, Caregiver, and Educator. The search terms for **concept** included: Drug Knowledge, Medication Knowledge, Rational Drug Use, Medication Literacy, Pharmaceutical Literacy, Medication Safety, Drug Safety, Popular Science, Health Education, Medication Education, Knowledge-Attitude-Practice (KAP), Medication Misconception, Medication Error, and Adverse Drug Reaction (ADR).

#### 2.1.4 Data extraction and synthesis

From June to July 2025, basic information of included studies (first author, publication year, country of study), background environment, research purpose, population characteristics (type of study population, age of study population), investigation methods, research content or items, influencing factors and etc. were extracted. In accordance with the Thematic Synthesis method (12), three-tiered inductive integration of potential popular science content from the included studies was carried out. Following the classic pathway of “free coding → induction of descriptive themes → generation of analytical themes”, second-level indicators were extracted and further integrated to generate first-level indicators. The above work was conducted back-to-back by two researchers, who each summarized and integrated the data to form descriptive themes and analytical themes, then exchanged opinions and finally reached an agreement.

### 2.2 Construction of the evaluation indicator system for pediatric pharmaceutical science popularization knowledge by the Delphi method

#### 2.2.1 Expert selection

Experts from tertiary hospitals and colleges of pharmacy/public health in the eastern, central, and western regions of China were selected, and the consultants were required to meet the following conditions: (1) engaged in pharmacy, pediatrics, clinical pharmacy and other related work;(2) with intermediate or higher professional titles; (3) working experience of more than 10 years.

#### 2.2.2 Questionnaire design

This study adopted a progressive technical pathway of “Literature Research → structured questionnaire → Delphi consultation”.

Since the research team had fully extracted the potential item pool in the field of pediatric medication science popularization and summarized the first-level and second-level indicators through preliminary literature research, the open-ended brainstorming session of the first round in the traditional Delphi method was omitted, and a structured questionnaire was directly developed. The questionnaire was designed with the Guidelines for Quality Control of Medical Delphi Studies (2022 Edition) as the methodological reference, and was divided into the following four parts: (1) Basic information and professional background of experts, including age, professional title, educational background, working years, professional field, etc.; (2) Self-assessment of expert authority; (3) Importance evaluation of first-level indicators and collection of open-ended comments; (4) Importance evaluation of second-level indicators and collection of open-ended comments. The importance of indicators was scored using a 5-level Likert scale: 1 = very unimportant, 2 = relatively unimportant, 3 = moderately important, 4 = relatively important, 5 = very important. An open-ended text box was set after the scoring area of each indicator to collect experts' suggestions on modification or adjustment of the indicator.

To ensure the operability and content validity of the questionnaire, a pilot survey was conducted with 3 experts in the field of pharmacy who were not involved in the questionnaire development prior to official distribution. The experts evaluated the item wording, logical structure and completion time of the questionnaire, and minor adjustments were made to the questionnaire wording based on the feedback to finalize the official version.

#### 2.2.3 Consultation steps and decision criteria

Based on the established structured questionnaire, the open-ended brainstorming session of the first round in the traditional Delphi method was omitted in this study. The single-round distribution and collection of electronic questionnaires were completed via the Wenjuanxing platform in September 2025, and expert evaluation was conducted using a highly structured 5-level Likert scale. A dual-threshold criterion was set as the item adoption standard: items with a mean score ≥ 4.0 and a coefficient of variation (CV) ≤ 25% were considered to have reached a consensus on importance among experts, and were directly adopted into the final indicator system. For items failing to reach consensus, the research team conducted joint deliberation in combination with qualitative revision suggestions submitted by experts in the open-ended feedback section. Such items would either be directly deleted, or substantially revised and merged at a lower level into relevant retained items.

### 2.3 Statistical methods

Excel and SPSS 25.0 were used for statistical analysis. The questionnaire recovery rate (R) was used to evaluate the enthusiasm of experts, and R ≥ 85% was considered as good enthusiasm. The authority of experts was expressed by the authority coefficient (Cr), which was quantitatively calculated from experts' familiarity with the indicators (Ca) and judgment basis (Cs), with the formula Cr = (Ca + Cs)/2. A Cr value ≥ 0.70 indicated a high degree of expert authority. The importance scores of indicators were described by “mean ± standard deviation ($\bar{x}$±s)”; the screening criterion was set as: $\bar{x}$≥4.00 (based on the 5-level Likert scale), which was regarded as concentrated expert opinions and high importance of the indicator. The consistency of expert opinions was evaluated by the intraclass correlation coefficient (ICC) and coefficient of variation (CV). An ICC ≥ 0.75 was defined as excellent, 0.60–0.74 as good, 0.40–0.59 as moderate, and < 0.40 as poor; a CV ≤ 25% was considered acceptable dispersion of expert opinions. Cronbach's α coefficient was used to evaluate the reliability of the questionnaire, and an α ≥ 0.70 indicated acceptable reliability, α ≥ 0.80 indicated good reliability; item-level content validity index (I-CVI) ≥ 0.78 indicated good item validity, and scale-level content validity index (S-CVI) ≥ 0.90 indicated good overall scale validity. The test level was set at α = 0.05.

## 3 Results

### 3.1 Literature search results

The screening followed the PRISMA workflow. Of 3,520 initial studies, 2,850 remained after deduplication. After title/abstract screening, 125 full texts were assessed, and 68 were finally included (Table 1).

**Table 1:** Literature screening process and results.

Procedure	Process Steps	Specific Content	Number (n)
Identification	Records identified via databases	SinoMed, VIP Network, Wanfang Data, CNKI, PubMed	2,510
	Records identified via other sourcess	National Standard Information Public Service Platform, National Standard Library, CNKI Standard Library, ISO, ANSI, European Standards, ASHP	1,010
Abstract Screening	Duplicate records removed	Duplicate publications, duplicately included reviews	670
	Records excluded after title and abstract screening	Exclusion of non-empirical or non-research literature, including policy comments, news reports, conference notices, call for papers, product introductions, drug instructions, and exclusion of irrelevant studies	2,635
	Records excluded due to unavailability of full text or language restrictions	—	72
Full-text screening	Full-text articles assessed for eligibility	Excluded articles with off-topic content, studies lacking survey data, duplicate publications, and study protocols	57
Included	Studies included in the final review	—	68

### 3.2 Preliminary framework indices via thematic synthesis

A total of 892 content items were extracted from the included studies, 54 s-level indicators were summarized, and finally integrated into 11 first-level indicators, and a preliminary evaluation indicator system for pediatric pharmaceutical science popularization knowledge was formulated.

### 3.3 Construction of the evaluation indicator system for pediatric pharmaceutical science popularization knowledge

#### 3.3.1 Questionnaire response rate

In the single round of expert consultation, 28 questionnaires were distributed, and 26 were returned (R = 92.86%). The experts demonstrated good willingness to participate.

#### 3.3.2 Expert demographics

The 26 invited experts were from eastern, central, and western China. Their workplaces included tertiary hospitals, colleges of pharmacy/public health, etc. Detailed expert demographics are presented in Table 2.

**Table 2:** Basic information of experts (*n* = 26).

Item	Category	Number (n)	Ratio (%)
Regional distribution	Eastern China (Beijing, Shanghai, and Jiangsu)	8	30.8
	Central China (Hubei, Hunan)	7	26.9
	Western China (Sichuan, Chongqing, and Yunnan)	11	42.3
Workplace	Tertiary hospital	15	57.7
	College of pharmacy/public health	11	42.3
Professional title	Chief physician/Chief pharmacist	3	11.5
	Deputy Chief Physician/Deputy Chief Pharmacist	13	50.0
	Pharmacist-in-Charge/Attending Physician	8	30.8
	Others	2	7.7
Professional field	Pharmacy (Clinical Pharmacy/Pharmaceutics)	13	50.0
	Pediatrics	7	26.9
	Public Health/Health Education	6	23.1
Work years (years)	10–15	7	26.9
	16–20	11	42.3
	>20	8	30.8

The expert authority coefficient (Cr) was 0.75 (Ca = 0.91, Cs = 0.60), indicating that the degree of expert authority was **acceptable**.

#### 3.3.3 Importance scoring and consistency of indices

The mean importance scores of the 11 first-level indicators rated by experts ranged from 3.49 to 4.67. The intraclass correlation coefficient (ICC) of each indicator ranged from 0.360 to 0.938 (all *P* < 0.05). Indicators with ICC at moderate or higher levels accounted for 90.9% of all indicators, indicating **generally good** consistency of expert opinions. The coefficient of variation (CV) of all indicators was ≤ 25%, and the dispersion of expert opinions was acceptable.

According to the dual-threshold criterion (mean score ≥ 4.0 and CV ≤ 25%), 6 indicators were directly retained, namely Introduction to Drugs, Medication Safety, Medication Misconceptions, Household Medication Guidelines, Pharmacist Functions and Legendary Stories of Drugs. Although *in vivo* Drug Process had a slightly lower mean score (mean = 3.91), it was retained after the research team's deliberation combined with experts' qualitative comments. The mean scores of **Prescription Reading**, **Drug Efficacy and Toxicity**, **Drug Use** and **Drug Economy and Regulation** were all below 4.0. Combined with experts' qualitative feedback (such as “abstract concepts” and “exceeding children's cognitive load”), the research team decided after collective discussion to exclude the two indicators Prescription Reading and Drug Economy and Regulation. The relevant content of Drug Efficacy and Toxicity was merged at a lower level into the second-level entries of Medication Safety, while the operational content of Drug Use was merged at a lower level into the second-level entries of Household Medication Guidelines. The importance scores and consistency status of each indicator are shown in Table 3.

**Table 3:** Evaluation of each dimension in expert consultation.

Index Category	$\bar{x}$± s	CV (%)	I-CVI	ICC (95% CI)	*P*-value	Cronbach's *α*
Drug Introduction	4.27 ± 0.68	15.95	0.98	0.732 (0.532–0.864)	<0.001	0.75
*In Vivo* Drug Process	3.91 ± 0.80	20.56	0.92	0.527 (0.352–0.708)	<0.001	0.85
Medication Safety	4.38 ± 0.66	15.20	1.00	0.503 (0.327–0.690)	<0.001	0.84
Misunderstandings in Medication	4.37 ± 0.69	15.87	1.00	0.532 (0.392–0.695)	<0.001	0.93
Home Medication Guide	4.33 ± 0.71	16.38	1.00	0.730 (0.597–0.846)	<0.001	0.94
Prescription Reading	3.79 ± 0.83	21.85	0.94	0.360 (0.118–0.605)	0.001	0.63
Drug Efficacy and Toxicity	3.80 ± 0.86	22.53	0.90	0.642 (0.466–0.795)	<0.001	0.88
Drug Use	3.86 ± 0.87	22.47	0.96	0.431 (0.190–0.659)	<0.001	0.70
Pharmacist Functions	4.67 ± 0.56	11.89	1.00	0.938 (0.866–0.972)	<0.001	0.97
Legendary Stories of Drugs	4.12 ± 0.77	18.79	1.00	0.784 (0.662–0.881)	<0.001	0.95
Drug Economy and Regulation	3.49 ± 0.74	21.24	0.92	0.518 (0.325–0.708)	<0.001	0.81

#### 3.3.4 Questionnaire reliability and validity

The Cronbach's *α* of this questionnaire ranged from 0.63 to 0.97, among which the *α* coefficients of 10 dimensions were all greater than 0.70, indicating that the overall scale had acceptable to good internal consistency reliability. Among them, the Cronbach's α of Prescription Reading was 0.63. Combined with its failure to meet the consensus threshold and experts' qualitative feedback, this indicator was excluded.

The item-level Content Validity Index (I-CVI) of all second-level indicators ranged from 0.90 to 1.00, all higher than the acceptance standard of 0.78; the scale-level Content Validity Index/Average (S-CVI/Ave) was 0.96, exceeding the recommended standard of 0.90, indicating excellent content validity of the overall scale.

#### 3.3.5 Revision results of second-level indicators

For the 54 initial second-level indicators, the research team deleted 16 entries of abstract concepts, added 8 new entries including Drug Prices and Patents and Biological Product Storage, and merged and downgraded relevant second-level entries under Drug Efficacy and Toxicity and Drug Use based on the dual-threshold statistical results and experts' qualitative feedback. The final indicator system consists of 7 first-level indicators and 46 s-level indicators.

## 4 Discussion

### 4.1 Scientificity of the study design

Research in this field requires both pharmaceutical expertise and compliance with the laws of children's cognitive development, which cannot be fully covered by experts from a single discipline. Under this premise, the open first round of the traditional Delphi method would prevent experts from generating age-appropriate popular science entries independently due to insufficient interdisciplinary knowledge. This study constructed the evaluation indicator system following the pathway of “systematic literature review—thematic synthesis—single-round modified expert Delphi consultation”. In the preliminary stage, a sufficient initial item pool and structured questionnaire were formed based on saturated retrieval and thematic induction of 68 pieces of literature. By omitting the open-ended inquiry of the first round in traditional Delphi and directly adopting a structured 5-level Likert questionnaire for single-round expert evaluation, the study achieved a balance between methodological efficiency and content integrity (13). A dual validation mechanism of “statistical threshold anchoring + qualitative feedback calibration” was implemented for indicator screening: indicators that failed to meet the threshold were processed hierarchically in combination with experts' open-ended comments. This mechanism not only ensures the objective measurability of consensus, but also bridges the adaptation gap between statistical thresholds and real popular science scenarios through qualitative feedback (14).

### 4.2 Analysis of Low-consensus indicators

Abstract microscopic concepts such as receptor theory, transporters and metabolic enzymes were deleted to avoid excessive extraneous cognitive load that hinders schema construction, which is consistent with Piaget's definition that children aged 7–12 are in the concrete operational stage (15). Children at this stage still rely on concrete objects and intuitive representations to develop logical thinking. Forcing the introduction of abstract terms will cause excessive extraneous cognitive load and further impede the schema construction of core concepts (16–18). The removal of content detached from daily scenarios such as medication dosage abbreviations avoids redundant rote memorization, which aligns with the concept that science education for young children should focus on core concepts and avoid content beyond their cognitive level (19, 20). Differences in expert consensus exist across dimensions: the ICC of Prescription Reading is 0.360, and that of Drug Economy and Regulation is 0.431, reflecting cognitive divergence between clinical practitioners and public health scholars. The former advocates the popularization of professional medication skills, while the latter worries about age-appropriateness risks caused by abstract concepts. This divergence is not a methodological flaw; on the contrary, it confirms the difficulty of balancing “professionalism” and “accessibility” in pediatric pharmaceutical popular science. Different from direct deletion, Drug Efficacy and Toxicity and Drug Use, though of clinical importance, were downgraded to second-level entries as their scores failed to meet the inclusion threshold for first-level indicators. Expert review confirmed that setting them as independent chapters would not only increase extraneous cognitive load through abstract pharmacological concepts such as the duality of “efficacy-toxicity”, but also logically fall under the categories of medication safety and home care, leading to content redundancy if set as separate chapters.

### 4.3 Pedagogical logic and literacy cultivation dimensions of core indicators

The optimization of the indicator system is closely centered on cognitive load management. According to the analysis of questionnaire feedback from experts, among the 7 finally retained first-level indicators, 4 indicators meet the criteria of mean score ≥ 4.0, ICC ≥ 0.7 and Cronbach's *α* ≥ 0.7 simultaneously, namely Introduction to Drugs, Pharmacist Functions, Household Medication Guidelines and Legendary Stories of Drugs. As priority indicators in the system (21), these four systematically correspond to literacy cultivation goals of different dimensions, forming a complete closed loop from cognitive foundation to social participation, and from family practice to scientific spirit. Introduction to Drugs guides children to abstract the general properties and classification logic of drugs from specific drug cognition, laying the foundation of scientific cognitive literacy (22). Pharmacist Functions emphasizes clarifying the credible role of professionals, cultivating children's health information literacy and social role cognition (23). The content of household standing drug management transforms pharmaceutical knowledge into operable life skills, shaping awareness of family health management and safety practice literacy (24). The origins of world-changing drugs such as artemisinin and metformin carry scientific humanistic literacy and innovation awareness, guiding children to perceive the interactive relationship between science, technology and social development (25).

Based on experts' qualitative revision suggestions and collective deliberation, the research team transformed the complex ADME process of “*in vivo* Drug Process” into “adventure stories inside the body”, successfully converting intrinsic cognitive load that may hinder learning into germane cognitive load that promotes schema construction, which embodies the concept of scaffolding instruction (26, 27). Meanwhile, new second-level indicators such as Drug Prices and Patents and Biological Product Storage were added to help children establish a preliminary understanding of the cost of health innovation and social ethics, bridge the gap between “knowledge” and “practice”, and advance popular science education from theoretical cognition to the family practice scenario closely related to life safety (28, 29).

### 4.4 Bridging the translational gap: narrative and gamification strategies

The construction of the knowledge system is only the first step; how to effectively transmit it to children is a greater challenge. This study is committed to bridging the “transformation gap” through two strategies. First, promoting the “narrative turn” strategy. We advocate not directly teaching “pharmacokinetics”, but telling “the journey of drugs”; not saying “adverse reactions”, but introducing “uninvited guests”. This metaphorical narrative, which compares the immune system to an “army” and antibiotics to “guided missiles”, conforms to children's cognitive way of understanding the world through stories. Studies have shown that narratives can more effectively activate the logical and emotional centers of children's brains and promote deep learning (30, 31). Second, adopting “gamified risk simulation”. Retaining scenario simulations such as “blindly believing in imported drugs” is not for preaching, but designing them as “errors to be avoided” scenarios. This method enhances children's risk perception through creating personal experience, conforms to the law that “consciousness raising” is the first step of change in the Transtheoretical Model of Behavior Change (32) and sows the seeds for the establishment of future health behaviors.

### 4.5 Limitations

This study has the following limitations. **(1)** The expert panel lacks specialists in developmental child psychology, cognitive psychology and frontline primary school educators. To compensate for this deficiency, the initial item inclusion was primarily based on the research team's literature synthesis and extraction rather than direct assessment by single-domain experts. In addition, during the compilation of the popular science book, the team has invited primary school educators to participate in the content readability review, and the planned campus lectures have a built-in children's comprehension feedback module. These implementation data will provide an empirical basis for subsequent indicator revision with the involvement of educational psychology experts. **(2)** The fine balance of “age appropriateness” is a major challenge, as some concepts in dimensions such as *in vivo* Drug Process may still be abstract for young children. **(3)** How to balance “scientific rigor” and “interesting appeal” is a long-term challenge. **(4)** Evidence of intervention efficacy is lacking. The actual impact of this indicator system on students' knowledge, attitudes and behaviors remains to be verified by randomized controlled trials (RCTs) or pre-post test questionnaire studies. **(5)** The indicator system of this study takes children as the direct knowledge recipients, and its impact on guardians is mainly realized through the “reverse socialization” effect after children's cognition is improved. Future studies can supplement and develop an independent evaluation module for medication popular science targeting guardians on this basis.

## 5 Conclusion

In conclusion, this study established a pediatric pharmaceutical science popularization indicator system via the Delphi consensus method and conducted age-appropriate refinement in accordance with cognitive theories. The general popular science books on pediatric pharmacology compiled based on this indicator system have been finalized, and a series of science popularization lectures for primary school campuses have been planned. However, its ultimate value hinges on whether it can effectively spark children's interest in pharmaceutical knowledge, translate professional content into accessible health cognition, and provide a reusable content blueprint for the development of standardized pediatric pharmaceutical science popularization resources.

Future research should focus on the following directions: (1) Supplement perspectives of child cognition and educational practice, engage child psychology experts and front-line teachers, and carry out iterative validation and age-appropriate revision of the indicator system by incorporating feedback on children's comprehension from campus lectures; (2) Explore multimodal teaching strategies, and reduce the cognitive load of abstract concepts through visual scaffolds such as augmented reality (AR) and physical models (33); (3) Conduct empirical effect evaluation, develop educational intervention programs based on this framework, and verify their short-term and long-term impacts on children's medication knowledge, safety attitudes and health behavioral intentions via randomized controlled trials.

## Data Availability

The datasets presented in this study can be found in online repositories. The names of the repository/repositories and accession number(s) can be found in the article/Supplementary Material.
